# Mobile-health tool to improve maternal and neonatal health care in Bangladesh: a cluster randomized controlled trial

**DOI:** 10.1186/s12884-018-1714-4

**Published:** 2018-04-16

**Authors:** Ruoyan Gai Tobe, Syed Emdadul Haque, Kiyoko Ikegami, Rintaro Mori

**Affiliations:** 1 Department of Health Policy, National Center for Child Health and Development, Tokyo, Japan; 2 UChicago Research Bangladesh (URB), Upazila, Bangladesh; 3 School of Tropical Medicine and Global Health, Nagasaki University NCGM Satellite, Tokyo, Japan

**Keywords:** Cluster randomized trial, Bangladesh, Mobile health network, Home-based health record, Maternal and neonatal care, Rural community

## Abstract

**Background:**

In Bangladesh, the targets on reduction of maternal mortality and utilization of related obstetric services provided by skilled health personnel in Millennium Development Goals 5 remains unmet, and the progress in reduction of neonatal mortality lag behind that in the reduction of infant and under-five mortalities, remaining as an essential issue towards the achievement of maternal and neonatal health targets in health related Sustainable Development Goals (SDGs). As access to appropriate perinatal care is crucial to reduce maternal and neonatal deaths, recently several mobile platform-based health programs sponsored by donor countries and Non-Governmental Organizations have targeted to reduce maternal and child mortality. On the other hand, good health-care is necessary for the development. Thus, we designed this implementation research to improve maternal and child health care for targeting SDGs.

**Methods/design:**

This cluster randomized trial will be conducted in Lohagora of Narail District and Dhamrai of Dhaka District. Participants are pregnant women in the respective areas. The total sample size is 3000 where 500 pregnant women will get Mother and Child Handbook (MCH) and messages using mobile phone on health care during pregnancy and antenatal care about one year in each area. The other 500 in each area will get health education using only MCH book. The rest 1000 participants will be controlled; it means 500 in each area. We randomly assigned the intervention and controlled area based on smallest administrative area (Unions) in Bangladesh. The data collection and health education will be provided through trained research officers starting from February 2017 to August 2018. Each health education session is conducting in their house. The study proposal was reviewed and approved by NCCD, Japan and Bangladesh Medical Research Council (BMRC), Bangladesh. The data will be analyzed using STATA and SPSS software.

**Discussion:**

For the improvement of maternal and neonatal care, this community-based intervention using mobile phone and handbook will do great contribution. Thus, a developing country where resources are limited received the highest benefit. Such intervention will guide to design for prevention of other diseases too.

**Trial registration:**

UMIN000025628 Registered June 13, 2016.

## Background

Every year, approximately 290,000 women die due to pregnancy and childbirth, and 99% of maternal mortality occurring in the developing world. Bangladesh is one of those developing countries with high maternal mortality ratio (MMR), standing at 170 per 100,000 live births [1]. Although it has become a lower-middle income country with economic growth and achieved various targets of the Millennium Development Goals (MDGs), the target on reduction of maternal mortality and utilization of related obstetric and reproductive services provided by skilled health personnel remains unmet, and the progress in reduction of neonatal mortality ratio (NMR) lag behind that in the reduction of infant and under-five mortalities, with the proportion of neonatal mortality accounting for 39% of under-five mortality in 1991 increasing up to 60% in 2012 [2]. Reduction of maternal and neonatal mortality therefore remains as a crucial issue for the Sustainable Development Goals (SDGs), which aims to reduce MMR to less than 70 per 100,000 live births and NMR to less than 12 per 1000 live births in all over the world [3].

Accessibility to good-quality health care at both community and referral level is crucial to reduce maternal and neonatal deaths. In rural Bangladesh, where the home delivery by traditional birth attendants accounts for approximately 85% of all child birth [4], antenatal care visits as recommended by the World Health Organization (WHO), childbirth attended by skilled health personnel, and timely referral for emergent maternal and neonatal complications urgently need to be improved. As proved in previous studies, a participatory community-based intervention is effective to improve birth outcomes with low-cost, and realistic to achieve the progress for the rural area [5, 6].

In our proposed community-based intervention, we will test the effectiveness of two essential instruments: mobile phone and home-based maternal records. Mobile phone use is universal in Bangladesh, with the overall coverage of 98% in the population [7]. The costs of text message and audio call are relevantly low. Even in the rural area, where the fixed phones are much less available, mobile phone ownership has widespread and is expected to serve as a potential platform to implement interventions for a continuum of care throughout pregnancy, delivery, and postpartum as highlighted by the WHO [7]. Major effects of a mobile-health program include boosting communications between pregnant women and healthcare providers at different levels, recording their health status for a continuum of care, and providing necessary knowledge, information and guidance to users. It has been increasingly used into various health projects in developing countries [7–12]. A typical home-based health records for mothers and babies, the maternal and child health (MCH) handbook is an effective tool to facilitate client-provider communication and information-sharing, to record health status, to raise health awareness, to identify maternal and neonatal complications, and to encourage health-seeking behaviors [13]. Previous studies in several developing settings indicated its effectiveness to improve health seeking behaviors during pregnancy and postnatal periods [14–18]. On the other hand, its weaknesses are inconvenience to those illiterate, difficulty to preserve the data, and that loss or forgetting to take sometimes occurs. One of the major solution to improve the paper-based MCH handbook, the digitization by an application in a smart phone, an iPad or a desk-top, isn’t realistic in rural Bangladesh, as the coverage of both those electronic tools and the internet are relevantly low. Therefore, we attempt to incorporate the MCH handbook with the mobile short message and audio call, with the rationales that the MCH handbook facilitates the continuum of care throughout pregnancy, delivery and postpartum and has a potential to serve as the essential materials for mobile messages, with a hypothesis that such the intervention will be effective to improve utilization of health care and lead to better maternal and neonatal outcomes. Thus, the objective of the proposed study is to assess the effectiveness to improve birth outcomes and utilization of essential cares in rural Bangladesh, aiming to inform policy making for maternal and neonatal health to achieve the related targets in SDGs.

## Methods

### Study sites and population

This community-based cluster randomized controlled trial (RCT) will be conducted in two Upazilas (sub-districts) in Bangladesh. These two Upazilas are Dhamrai (from Dhaka District in Dhaka division) and Lohagora (from Narail District in Khulna division). Dhamrai has 16 Unions and Lohagora has 12 Unions (the smallest administrative unit in rural Bangladesh). Each Union has 20-25 villages with an approximate population of about 15,000 to 30,000. The rural population in Dhamrai and Lohagora is 306,704 and 215,902, respectively, with an annual birth rate of approximately 20 per 1000 [19]. Although local people supposed to get health care from the Union health care center but the reality is different due to lack of resources. Main source of income in the two study sites is agriculture. And in both areas participants are almost in the same level of education and socio-economically close. There was no related invention or health program during past five years that potentially influence the impact of our proposed study in the two settings. In each Upazila, there is one Upazila health complex and several health and family welfare centers and satellite clinics are located in unions and village level Mobile users are common in the respective areas with a good network by the mobile companies.

**Fig. 1:**
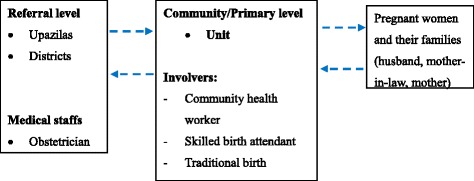
Study population and intervention

Our target population is pregnant women at reproductive age (15-49 years old). Eligible criteria include: 1. currently having a good health status, without any maternal complication, 2. living and planning to give a birth in the study settings during the period from February 01, 2017 to August 30, 2018 and 3. willing to participate to the proposed study with agreement to the informed consent. The study population is an open cohort: those target women will be invited to enter the study when they are detected to be pregnant. Our research team is explaining the procedure of the study and participant’s involvement and if the participants are agreed then received the written consent from the participants of this study. Participants also include community health workers (CHWs), skilled birth attendants (SBAs), staffs from community clinic and support committee for community clinic at the community / primary level. To facilitate referral and consultants from those staffs from the primary level, we will include health professionals working at healthcare facilities of the referral level (Upazilas, districts) to cooperate our intervention. Figure 1 shows the image of our proposed intervention.

### Randomization and masking

We will include all Union in the two Upazilas except the *sadar* Union because of its suburban status and better socio-economic status. There are 16 unions in *Dhamrai* and 12 unions in *Lohagora*. To avoid potential contamination, the cluster sampling target will be Unions, rather than the individual. In general, Unions in the targeted study sites have homogeneous socioeconomic characteristics such as income level of household, annual birth rate, accessibility to primary healthcare, literate rate, school attendance, and hygiene conditions in household.

**Fig. 2:**
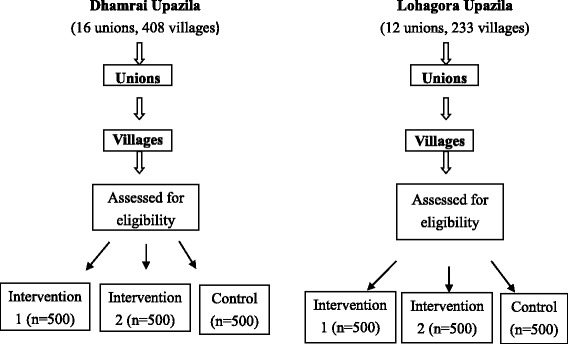
Randomization and allocation

Figure 2 is the process of randomization and allocation. Based on the required sample size as described below and population in each Upzila, a total of 12 Unions (6 Unions in *Dhamrai* and 6 Unions in *Lohagora*) are selected randomly and allocated to either the intervention or the control. Project staffs affiliating to the NGO in charge of this study will implement this sampling and allocation process. First, in each Upazila, cluster names will be written on paper and folded and put into an opaque bottle. In the first round, the clusters joining the proposed study will be randomly selected. Then, by the same method, 6 from the 17 clusters in *Dhamrai* and 6 from the 12 Unions in *Lohagora* will be randomly selected and assigned to the intervention, and the last cluster will be assigned to the control. In the selected clusters, all eligible pregnant women are subject to the recruitment. We will obtain a full list of pregnant women living in the selected Unions from Upazila health complex collected by their staffs and visit household of those pregnant women by the project staffs. The project staffs are experienced to work in the field and they are trained for this project. Due to the natural characteristic of the intervention, all participants, including pregnant women and their families, staffs at the community / primary level and health professionals at the referral level enrolled the study cannot be masked to their study group allocation. Our field staffs are either involved in the sampling and allocation process or inferred from the recruitment and the field survey during the study. Rather, staffs in charge of data input will be blinded to the allocation.

### Intervention

In the intervention settings, we plan to fully apply the platform of mobile short messaging and audio system and to effectively link pregnant women and their family members, CHWs and other involved in maternal and perinatal care at the primary level (village and union), and health professionals at the referral level (Upazila and District). Such a network is used to provide information and health education and to boost communication among various stakeholders. The contents of the existing version of Bangladeshi MCH handbook will be incorporated into the mobile platform, with short message or audio message edited based on the gestational age and individual needs. The mobile platform is expected to strengthen the application of MCH handbook. We also are providing books to the participants for each in the intervention areas.

The primary study target is pregnant women. Their families (husbands, mother-in-law and / or mothers) are also invited to participate to the intervention, as they are often the key person to determine daily health-related behaviors, home-based care during and after pregnancy, and seeking and utilization of healthcare services during pregnancy, childbirth and neonatal period in rural Bangladesh. By using the network, pregnant women and their families are possible to daily consult and seek advices during pregnancy with CHWs and to seek basic childbirth and postnatal services to village doctors and SBAs. Meanwhile, it is also possible to realize communications among health care providers at different levels: for example, CHWs, village doctors and SBAs can consult and seek advices and make referral of maternal complications to health professionals at the upper-level facilities (Upazila and District), and health professionals may contact the primary / community-level staffs for the effective referral and provide necessary advices and training as well. As CHWs play an important role to provide primary healthcare to residents in the village community, our trained field staffs will work together with CHWs to take care of pregnant women in intervention group. By integrating CHWs, SBAs and village doctors at the primary level and health professionals at the referral level, the network will also provide advices on a continuum of care to identify complications or any condition needing referral and to alert and help to seeking ANC, SBAs and facility-based healthcare services including emergency care when necessary to pregnant women and their families. As another effective tool to collect data, the MCH handbook will be recorded by CHWs at the primary level, to boost communication between pregnant women and healthcare providers and to implement health education.

This study has been approved by the ethical committee of Bangladesh Medical Research Council, Bangladesh and National Center for Child Health and Development in Japan. We don’t expect the intervention to have adverse effects to the participants and the study sites. The informed content will be implemented targeting all participants, and all paper-based and electronic-based personal information will be anonymous in data analysis.

### Study procedures

The study period includes one-year recruitment of participants and follow-up during pregnancy and the neonatal period. The study starts from February 01, 2017 when we start to enroll the participants, and is expected to finish on August 30, 2018 when all participants go through the neonatal period, four weeks after giving birth. All eligible pregnant women living in the selected villages for the intervention and the control will be recruited right after the allocation. Our trained staffs will visit each household to collect the baseline and the end-of-point data on the pregnancy by using a structured questionnaire at the enrolment and the end of four weeks after delivery or cessation of pregnancy, the ending point of the participation, respectively. In the survey, variables related to the expected outcomes as describe below as well as demographical and socioeconomic characteristics and indicators on knowledge and practices will be obtained. A code to identify each participant during the study period will be created.

In the intervention group, a short message will be sent to the participants to welcome them and the printed version of MCH handbook will be provided to the participants to the intervention. Our trained staffs together with CHWs will build a close linkage with pregnant women and their family members by using mobile short messaging and audio system. To monitor their conditions and make a record, the staffs will send a short message to the participants at the frequency of every two weeks and to alert them to visit for antenatal care in advance, and if necessary, also visit their home to talk with the participants face-to-face. As the coverage of at least four visits of antenatal care recommended by WHO is low in rural Bangladesh, our first target will to improve the number of antenatal care visits. Then, text messages on essential health items including perinatal care, including antenatal care visit, nutrition during pregnancy, intake of iron tablet and folic acid, work, rest and daily activities, husband and families’ support during pregnancy and lactating period, danger signs, list of skilled birth attendants and hospitals, signs of labor, postnatal care, neonatal care, breastfeeding, and family planning, will be edited to be user-friendly and sent accordingly to each participant appropriate to their gestational age and location of the residence. For those cannot read, our staffs will use phone call instead of short messages. For those participants who don’t own a mobile, we will ask the neighbors or the relatives living closely to them to share the mobile where messages could be sent, because of the close liaison in the rural community. Every two month, the enrolled pregnant women and their families and CHWs will be organized to a community meeting by the mobile network, for face-to-face health education, consulting / advices and ANC during pregnancy. Data on health status during pregnancy, childbirth and postpartum, utilization of related healthcare services at community and facility levels, opinions of the participants and maternal and neonatal outcomes will be collected by using the mobile network and home visiting. On the other hand, in the control group, a routine care will be provided usually, but no intervention will be implemented. Related data will be collected by home visiting during the study period and the end-of-point survey.

### Measurement of outcomes

We don’t expect the intervention to have adverse effects at local and individual level. The primary outcome is neonatal mortality (deaths in the first 28 days per 1000 live-births), Secondary outcomes include maternal mortality (deaths of a pregnant women or within 42 days of cessation of pregnancy from any cause related to the pregnancy or its management, but not from accidental causes), stillbirth, miscarriage (cessation of a presumptive pregnancy before 28 weeks of gestation), preterm birth, low birth weight, maternal morbidities such as pregnancy complications or near miss as an alternative, frequency of antenatal care visits, accessibility to skilled birth attendants for delivery, referral for identified complications, utilization of postpartum care, and status of initiating breastfeeding. We will use International Classification of Diseases version 9 definition of stillbirth for the study settings [20].

### Quality control

A local NGO, Bridge of Community Development Foundation will take responsibility to maintain the quality of the implementation and data collection. The local Program coordinators will train the field staffs at the beginning of the project and provide enough education and refreshing training for the standard control as they are doing. The recruited community health workers, birth attendants in the community and field staffs will also receive refresh training from the maternal and child health experts and the health professionals working the referral level facility. They will get idea about their role in the community and necessary knowledge to provide essential care, identify the complications and implement referral to the upper-level facilities. At the end of the field study, we will provide an appropriate incentive to those participants who fully attend without any missing information, in order to reduce the drop-out and missing data and to express our thanks for their cooperation.

Data from the field study, including the questionnaire, the MCH handbook and other data collected from the study settings will be double-entered in an electronic database. The local project coordinators, local survey staffs and data managers will work together for the quality control.

### Statistical analysis

We assume annual birth cohort of 15 newborns in average in the village, a baseline neonatal mortality of 24.4 per 1000 in Bangladesh, a between-cluster coefficient of variation of 0.3, a 25% loss to follow-up, a type I error of 0.05, and statistical power of 0.80. Our study has a power of 40% to detect a 20% reduction in neonatal mortality at the 95% significance level. Thus, the total sample size is 2700 but we have added other 10% and we targeted the total sample is 3000.

First, univariate analysis will be performed to explore the characteristics of variables and the study population. In the comparison of each variable, a stratification by randomization groups will be implemented to examine the equality of covariates of the two groups at baseline. If a difference is found at baseline on one or more covariates, we will further adjust those covariates by using ratio residuals for each cluster obtained from a logistic regression model at the individual level at the cluster-level. Moreover, intention to treat analyses at village and participant levels will be implemented. Risk ratios (RR) for primary outcomes and secondary outcomes will be assessed and compared between the intervention group and the control group by using log-binomial regression. The results of RR will be presented with 95% CI. Additionally, considering a potential correlation in the expected outcomes within villages, a generalized estimating equations will be implemented, in which model village ID will be the subject variable. Options of the suitable working correlation structures such as independent, exchangeable, AR (1), M-dependent, and unstructured will be considered and the QIC statistics (the % QIC macro) will be used to select the final working correlation structure [21].

## Discussion

The intervention incorporating the home-based health records with the mobile health network aims to improve health seeking behaviors of pregnant women living in rural Bangladesh, for better maternal and neonatal health outcomes. The innovative intervention package, the combination of mobile phone network and MCH handbook can be relatively complemented and is expected to maximize both advantages in terms of knowledge dissemination, promotion of communication, preservation of home-based health records, and facilitation of continuum of perinatal care. To our knowledge, our proposed study will be the first one to measure the impact of such a combined approach. The cluster-randomized research design is robust to evaluate the effects of the proposed intervention and to provide insight to maternal and neonatal issues in the field.

We recognize that the high proportion of home-based delivery practiced by less skilled personnel in particular in the rural area remains as a bottleneck against achievement of the targets related to maternal and neonatal care in health-related SDGs, which figures are not comparative, but rather absolute. Therefore, as the fundamental step, to improve health seeking behaviors during pregnancy and postnatal period, especially utilization of antenatal care and skilled personnel and timely detection and referral in case of emergency is crucial for the achievement. Although tremendous efforts are necessary in both the demand and the supply side and there is still a long way towards achievement of related targets in SDGs, the project doesn’t have resources to improve service delivery intensively at health facilities, the supply side. Rather, the intervention focus on improving of utilization of antenatal care and skilled personnel, raising of health awareness of pregnant women and their families and strengthening the referral system by the mobile network, and effective link between the primary level and the referral level is expected to contribute to capacity building of CHWs and birth attendants.

In Bangladesh, the Ministry of Health and Family Welfare has started a nationwide project to increase health awareness by broadcasting text messages to all mobile phone numbers in 2007 [7]. On the other hand, the quality of the implementation and the impact of the SMS health messages on population health remain unknown. For pregnant women, while there is the mobile phone-based short message pregnancy advice system and those living remote villages are theoretically able to receive useful prenatal advice, the one-way operation may limit communication between healthcare providers in the community (CHWs and birth attendants) and pregnant women, and consequently the efficiency of the project. In other words, we concern that the potential of the mobile network may not be maximized. In our study, both text and audio messages will be fully applied to meet the individual needs and to boost communication among various stakeholders in the community. We take those don’t have mobile phone and those cannot read into consideration as well. A community-based approach, rather than a top-down governmental approach, will take the advantage of the close liaison of local members in the rural community of the country and assess the effectiveness of the intervention based on the scientific methodologies.

The study have some limitations due to relying on participants’ report, potentially causing bias of data. First, the pregnancy weeks will be reported by the participants as a precise detection by ultrasound is not available in the study settings. Moreover, cost data also rely on participants’ report. Health seeking behaviors depend on their husband’s decision. Nevertheless, by integrating and empowering the network at the communities, the proposal study is expected to improve health seeking behaviors including utilization of antenatal care and contribute to better maternal and neonatal health outcomes.

## Conclusions

This will be one of the first implementation study among women to improve the maternal and neonatal care using the mobile messages and home-based health record in Bangladesh, aiming to provide an innovative solution for MCH related SDGs. Know-how on the integration of mobile network and home-based health record would be disseminated and applied in other health program with successful experience.

## Abbreviations

ANC: Antenatal care
CHWs: Community Health Workers
MCH: Maternal and Child Health
MDGs: Millennium Development Goals
MMR: Maternal Mortality Ratio
NMR: Neonatal Mortality Ratio
RCT: Randomized Controlled Trial
SBAs: Skilled Birth Attendants
SDGs: Sustainable Development Goals
WHO: World Health Organization
